# Liquid Structure Scenario of the Archetypal Supramolecular Deep Eutectic Solvent: Heptakis(2,6-di-*O*-methyl)-β-cyclodextrin/levulinic Acid

**DOI:** 10.1021/acssuschemeng.3c01858

**Published:** 2023-06-02

**Authors:** Alessandro Triolo, Fabrizio Lo Celso, Sophie Fourmentin, Olga Russina

**Affiliations:** † Laboratorio Liquidi Ionici, Istituto Struttura della Materia-Consiglio Nazionale delle Ricerche (ISM-CNR), Rome 00133, Italy; ‡ Department of Physics and Chemistry, Università di Palermo, Palermo 90133, Italy; § Unité de Chimie Environnementale et Interactions sur le Vivant (UCEIV, UR 4492), Université du Littoral Côte d’Opale (ULCO), 59140 Dunkerque, France; ∥ Department of Chemistry, Sapienza University of Rome, Rome 00185, Italy

**Keywords:** supramolecular, hydrogen bonding, low melting mixtures, cyclodextrin, solvation

## Abstract

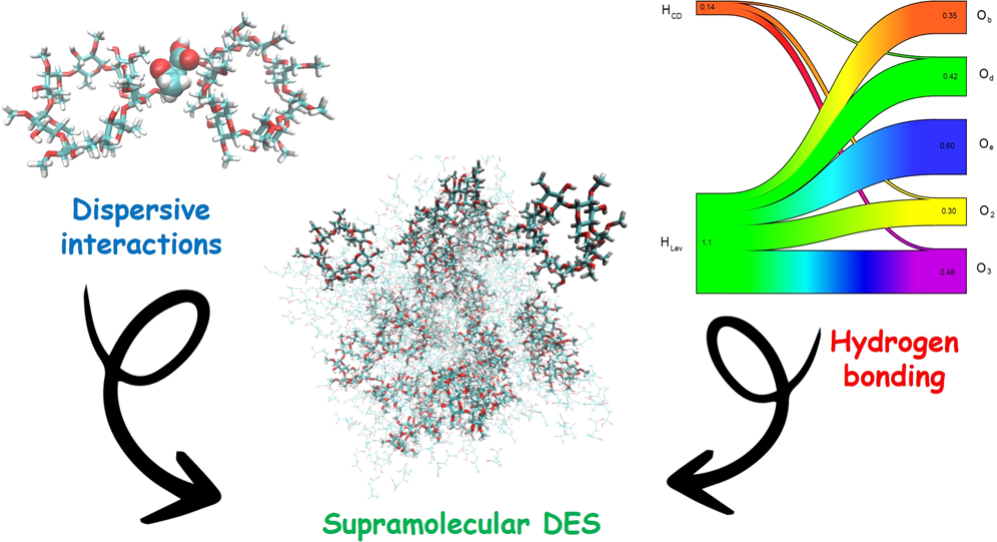

The concept of supramolecular solvents has been recently introduced, and the extended liquid-state window accessible for mixtures of functionalized cyclodextrins (CDs) with hydrogen bond (HB) donor species, e.g., levulinic acid, led to the debut of supramolecular deep eutectic solvents (SUPRA-DES). These solvents retain CD’s inclusion ability and complement it with enhanced solvation effectiveness due to an extended HB network. However, so far, these promising features were not rationalized in terms of a microscopic description, thus hindering a more complete capitalization. This is the first joint experimental and computational study on the archetypal SUPRA-DES: heptakis(2,6-di-*O*-methyl)-β-CD/levulinic acid (1:27). We used X-ray scattering to probe CD’s aggregation level and molecular dynamics simulation to determine the nature of interactions between SUPRA-DES components. We discover that CDs are homogeneously distributed in bulk and that HB interactions, together with the electrostatic ones, play a major role in determining mutual interaction between components. However, dispersive forces act in synergy with HB to accomplish a fundamental task in hindering hydrophobic interactions between neighbor CDs and maintaining the system homogeneity. The mechanism of mutual solvation of CD and levulinic acid is fully described, providing fundamental indications on how to extend the spectrum of SUPRA-DES combinations. Overall, this study provides the key to interpreting structural organization and solvation tunability in SUPRA-DES to extend the range of sustainable applications for these new, unique solvents.

## Abstract 2

X-ray scattering and molecular dynamics provide atomistic insight into the structural organization of an archetypal supramolecular deep eutectic solvent (SUPRA-DES) made of cyclodextrin and levulinic acid, providing ground for further sustainable applications.

## Introduction

Supramolecular chemistry is gaining growing interest in various research areas (e.g., food, medicine, environment).^1−3^ Host/guest chemistry is a branch of supramolecular chemistry, involving self-assembly and molecular recognition features;^4−6^ typically, properly sized and affine guest molecules get encapsulated either partially or entirely into the cavities of host molecules, leading to the formation of inclusion complexes, with remarkably improved physicochemical properties compared to naked guests. Among the various existing host molecules, natural or functionalized cyclodextrins (CDs) are the most studied one:^7^ the main reason is likely related to their green origin, wide natural availability, low price, and favorable molecular encapsulation characteristics. Native CDs are cyclic oligosaccharides, obtained from the enzymatic degradation of starch, that represent a class of readily available, harmless compounds with recognized biocompatibility. The most common representatives are α-, β-, and γ-CDs, comprising 6, 7, or 8 glucopyranose units, respectively, and possessing bucket-shaped cavities with an identical depth of ∼8 Å and inner diameters of ∼5.3, 6.5, and 8.3 Å, respectively.^8,9^ They possess a hydrophilic outer face prone to hydrogen bonding and a hydrophobic cavity that has been extensively exploited in food, cosmetic, and pharmaceutical applications to enhance the solubility for the reduction of the volatility or to improve the stability of guest compound by forming host/guest complexes. Such inclusion complexes have been extensively studied in aqueous solutions.^5,10,11^ On the other hand, few inclusion complexes have been described in other solvents or in the presence of cosolvent.^12−15^ It has been recently demonstrated that CDs could be efficiently solubilized in selected deep eutectic solvent (DES), such as the one based on choline chloride and urea^16−18^ and that, in such an environment, they retained their inclusion ability in this green solvent.^19−21^

DESs are a new generation of sustainable solvents composed of liquid mixtures of hydrogen bond acceptors (HBAs), often salts, and hydrogen bond donors (HBDs).^22,23^ The extended HB network that establishes upon mixing the two components leads to a drop in the melting point with respect to the ideal mixture one.^23,24^ DES properties allow their utilization in various fields like electrochemistry, organic synthesis, catalysis, extraction, separation processes, and pharmaceuticals.^25−27^ A platform of starting materials is available to form DESs, and so mixtures can be tailored to meet the requirements of a specific application. DESs can be prepared from nontoxic, widely accessible, cheap, and sustainable compounds^28^ including, as we have recently shown, CDs.^29−31^ Recently, El Achkar et al. explored the properties of binary mixtures of functionalized CDs (such as methylated β-CD) and HB active species such as levulinic acid (LevAc):^29,30^ such mixtures are composed of nonionic species, and no freezing temperature could be detected for them. Accordingly, a tentative proposal for new members of type V DES,^32^ so far indicated as supramolecular DES (SUPRA-DES), has been raised.^29,33^ In this respect, we notice that LevAc has been recently exploited as a component of other, more conventional, type III (e.g., refs (34−36)) and type V DES (ref (37)), where LevAc HB donor and/or acceptor ability has been perceived.

SUPRA-DES ability to improve the solubility of bioactive compounds was shown, and it was demonstrated that, while being part of the solvent, CDs retained their complexing ability.^30,38,39^ Still in their infancy, SUPRA-DES impact begins to be appreciated: Guo et al.^40^ exploited their association with polyoxometalates to develop functional supramolecular materials for energy and electronic applications; applications for extraction, absorption, and adhesive have been recently reported and reviewed.^33,41,42^ Considering the high SUPRA-DES compliance with the requirements of green chemistry, the observations collected so far are pointing toward new exciting applications of these CD-based solvents. Accordingly, a more profound comprehension of the interaction mechanism between the different components as well as the guest encapsulation process is necessary. Such issues were also mentioned in a recent review on CD-based deep eutectic supramolecular polymers.^43^

In this scenario, an atomistic level description of the solvation in the archetypal SUPRA-DES formed by heptakis(2,6-di-*O*-methyl)-β-cyclodextrin (DiMeβ-CD) and LevAc (at ratio 1:27) has been undertaken, aiming at rationalizing the interaction nature between the different components. We will make the synergic use of experimental scattering techniques and molecular dynamics simulations, an approach that proved already as a very powerful tool to access intimate structural details on DES.^44−46^ Overall, the study accounts for the atomistic level hierarchical architecture taking place in this SUPRA-DES: the mingling between hydrogen-bonding and dispersive interactions precludes CD aggregation and leads to a stable liquid phase with unaltered inclusion complex formation capability.

## Experimental and Computational

DiMeβ-CD was a CycloLab product (batch no.: CYL-4622), with purity >99% and an average degree of methyl substitution: 14.5. LevAc was a TCI product. Both components were kept under anhydrous conditions during storage and manipulation. The SUPRA-DES was prepared at a DiMeβ-CD/LevAc molar ratio equal to 1:27.^30^ The mixture was prepared by mixing the components in an inert atmosphere and subsequent stirring at 60 °C until a homogeneous limpid liquid was obtained.

Small-angle X-ray scattering (SAXS) measurements were performed at the SAXS Lab Sapienza with a Xeuss 2.0 Q-Xoom system (Xenocs SA, Sassenage, France), equipped with a micro-focus Genix 3D X-ray source (λ = 0.1542 nm) and a two-dimensional Pilatus3 R 300 K detector. The measurement covers the *Q* range: 0.1 Å^–1^ < *Q* < 3.2 Å^–1^. The sample was loaded into a disposable quartz capillary with a nominal thickness of 1.0 mm and sealed with hot glue. Measurements were conducted at an ambient temperature (ca. 20 °C), and standard background subtraction and data normalization were applied.

**Scheme 1:**
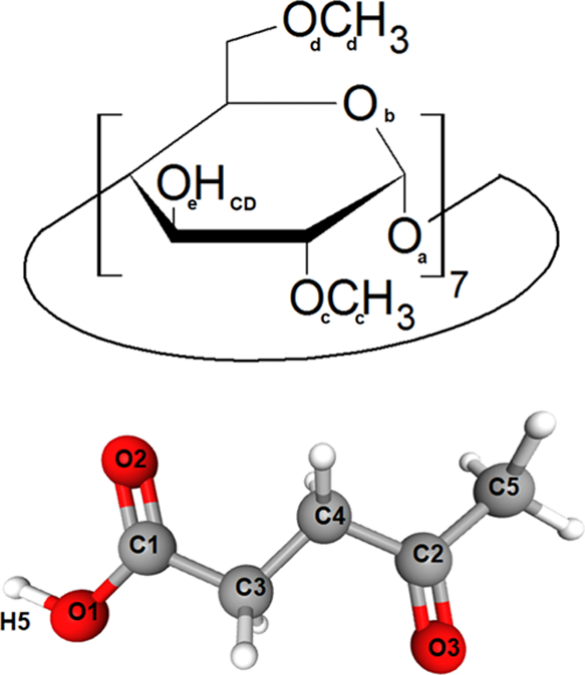
Schematic Representation of 2,6-Dimethyl β-Cyclodextrin (Top) and Levulinic Acid (Bottom), with the Nomenclature Used across the Publication

Molecular dynamics simulations were performed using the GROMACS 5.1.1 package.^47,48^ Concerning bonded and nonbonded parameters for the two components, LevAc (see Scheme 1), was described using an all-atom potential force field.^49,50^ DiMeβ-CD (see Scheme 1) was described using the q4-MD force field.^51−54^ Simulations were performed using a cubic box (box size ∼57 Å) containing 30 DiMeβ-CD molecules and 810 LevAc molecules and (1:27 molar ratio) (hereinafter indicated as the DiMeβ-CD–LevAc system); periodic boundary conditions were applied.

Initial configurations were created by Packmol software.^55^ The starting density was fixed 10% higher than the experimental one for LevAc. The equilibration procedure was done in several steps, starting from a 5 ns NVT simulation at 400 K, followed by a series of 5 ns NPT runs, lowering progressively the temperature (400, 350, and then 300 K) at 1 bar. After the equilibration phase, the system was run for a total of 100 ns for a production run, and then a trajectory of a further 2 ns was saved at a frequency of 1 ps for the calculation of the structural properties. The simulations were always checked versus the experimental density and the energy profile. During the production runs for the temperature coupling, we used a velocity rescaling thermostat^56^ (with a time coupling constant of 0.1 ps), while for the pressure coupling, we used a Parrinello–Rahman barostat^57^ (1 ps for the relaxation constant). The Leap-Frog algorithm with a 1.0 fs time step was used for integrating the equations of motion. Cutoffs for the Lennard-Jones and real-space part of the Coulombic interactions were set to 11 Å. For the electrostatic interactions, the particle mesh Ewald (PME) summation method^58,59^ was used, with an interpolation order of 6 and 0.08 nm of the FFT grid spacing.

X-ray and neutron-weighted structure factors were computed together with selected pair correlation functions, angular distribution functions, and spatial distribution functions using TRAVIS.^60−62^ The *gmx energy* routine of Gromacs was used to calculate two types of short-range potential: Lennard-Jones short-range (LJ-SR) and Coulombic short-range (Coul-SR). This utility routine was used on the final equilibrated trajectory where the different molecules (DiMeβ-CD and LevAc) were selected to obtain the partial energy contributions.

## Results and Discussion

The DiMeβ-CD–LevAc system has a very similar composition to the RAMEB–LevAc SUPRA-DES that was investigated by El Achkar et al. at the same molar ratio.^30^ RAMEB is a methyl-substituted β-CD, with a degree of substitution (DS) of ∼13, to be compared with DiMeβ-CD DS equal to 14.5. The RAMEB–LevAc system shows no crystallization events and is characterized by a glass temperature at −74.3 °C, hinting at a low melting mixture behavior for the system, thus addressing the classification as SUPRA-DES.^29^ Also, for the DiMeβ-CD–LevAc system, we could not detect a solidification even under prolonged maintenance at −10 °C. The RAMEB–LevAc system has been reported to be characterized by a density of 1.1845 g/cc at 303 K; our simulation on DiMeβ-CD–LevAc leads to a computed density of 1.2405 g/cc, with a deviation of <5%.

**Figure 1:**
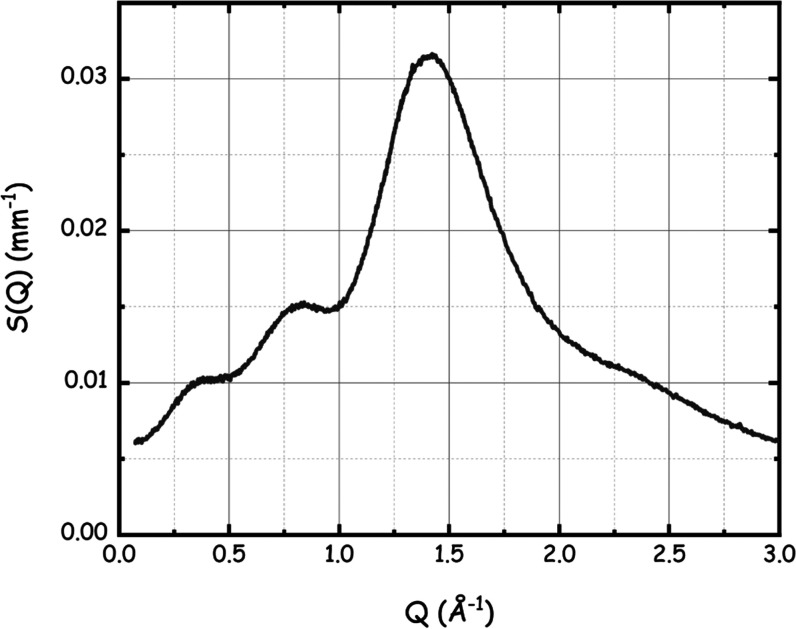
SAXS data from the DiMeβ-CD–LevAc SUPRA-DES system at ambient conditions.

**Figure 2:**
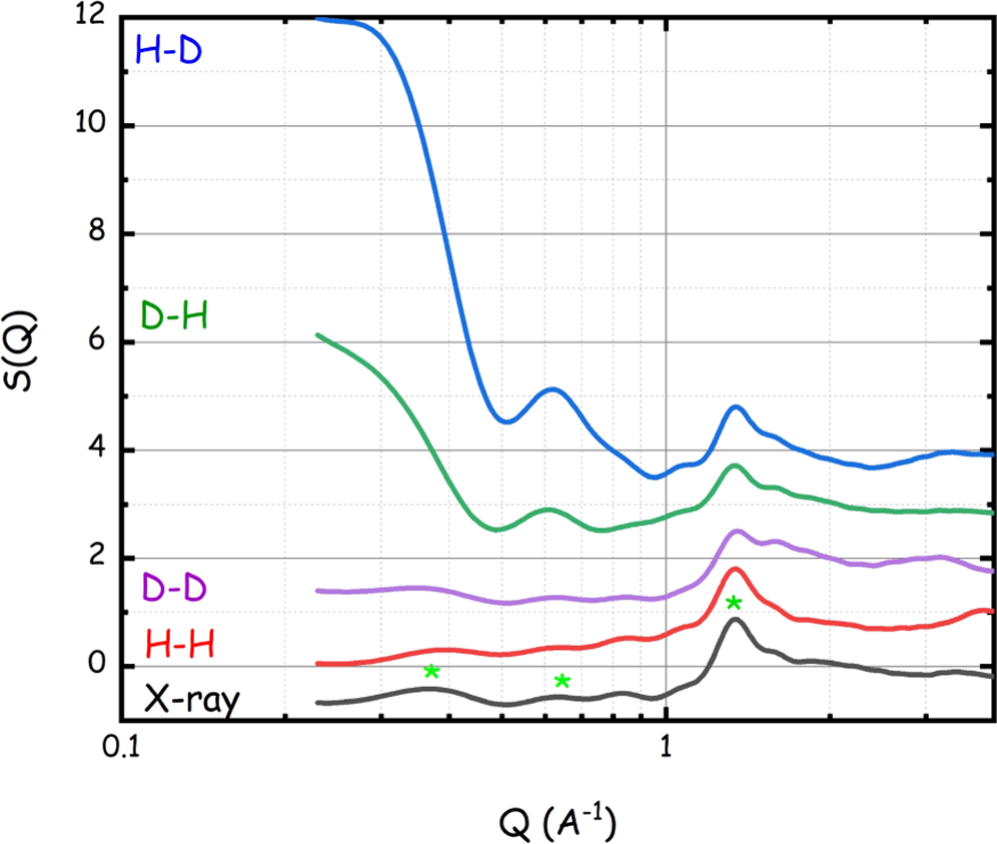
MD-computed X-ray-weighted (bottom) and neutron-weighted (top) data sets from the DiMeβCD–LevAc system at ambient conditions. Three peaks are highlighted in the X-ray pattern, referring to experimentally observed peaks at the same positions. The different neutron-weighted curves correspond to different selective deuteration situations as indicated in the plot, where the first letter and second letter refer to full protiated (H) or full deuterated (D) LevAc and DiMeβCD, respectively. Data have been vertically shifted for clarity.

The DiMeβ-CD–LevAc system was characterized by means of small-angle X-ray scattering (SAXS) in order to probe the mesoscopic organization of the mixture. The experimental SAXS pattern is shown in Figure 1: it is characterized by the presence of three clear peaks in the momentum range between 0 and 2.0 Å^–1^, which are centered at 0.37, 0.82, and 1.42 Å^–1^, respectively. Therein, it appears that the system is not characterized by long-range spatial correlations, as no indication of lower *Q* value scattering features appears. We notice that these data look different from related systems, such as the β-CD–reline system^18^ or the mixture of β-CD with the [C_2_mim][acetate] ionic liquid^63^ or even the solution of β-CD in water,^64^ where a clear indication of the isolated β-CD geometry (a hollow sphere, after isotropic averaging in space and time) could be detected, fingerprinting the unaggregated nature of β-CD dissolved in those media. The present experimental data set is compared with corresponding quantities extracted from MD simulations conducted on the same system. Figure 2 shows the computed X-ray-weighted (bottom curve) as well as several neutron-weighted *S*(*Q*)’s (corresponding to different selective deuterations of either the CD or the LevAc or both components) from the same simulated system. It appears that the simulated SAXS pattern (black, bottom curve in Figure 2) nicely accounts for the existence of peaks at 0.37, 0.83, and 1.34 Å^–1^ at positions equal to the experimental ones; peaks are highlighted in Figure 2 (note the log-lin scale in the figure). These features are maintained in the case of neutron scattering patterns from either both protiated or both deuterated components (red and violet curves in Figure 2). However, when selectively deuterating only one of the two components (either LevAc (green curve) or DiMeβ-CD (blue line)), a strong low *Q* signal (below 0.5 Å^–1^) manifests. This is the fingerprint of isolated DiMeβ-CDs, analogously to other CD solutions, where isolated CDs are homogeneously distributed,^18,63,64^ with a distinct feature at ca. 0.5 Å^–1^, as reported elsewhere.^18,63,64^ The mentioned difference between the SAXS pattern reported in Figure 1 and those found in other media^18,63,64^ is then the consequence of the small electronic density contrast between LevAc and DiMeβ-CDs in the present SUPRA-DES, which hinders the detection of the DiMeβ-CD form factor. Accordingly, the present SAXS pattern only reflects the structure factor associated with the neighbor CD interactions^64,65^ and the MD simulations clarify that, when properly choosing contrast (in the present case by the selective deuteration of either of the two components), neutron-weighted scattering reveals that the SUPRA-DES morphology corresponds to isolated CDs that are homogeneously distributed in the bulk, without intervening aggregation. Once rationalized the difference between the literature and present data sets, the reported SAXS pattern is then fully compatible with this structural model.

We further interrogated the MD simulations to extract structural information at the atomistic level on the DiMeβ-CD–LevAc system. Figure S1 shows a representative snapshot from the equilibrated MD simulation. It emerges that no collapsing of the DiMeβ-CD molecules can be detected, in agreement with the experimental observation that the present SUPRA-DES maintains homogeneous at ambient conditions.

**Figure 3:**
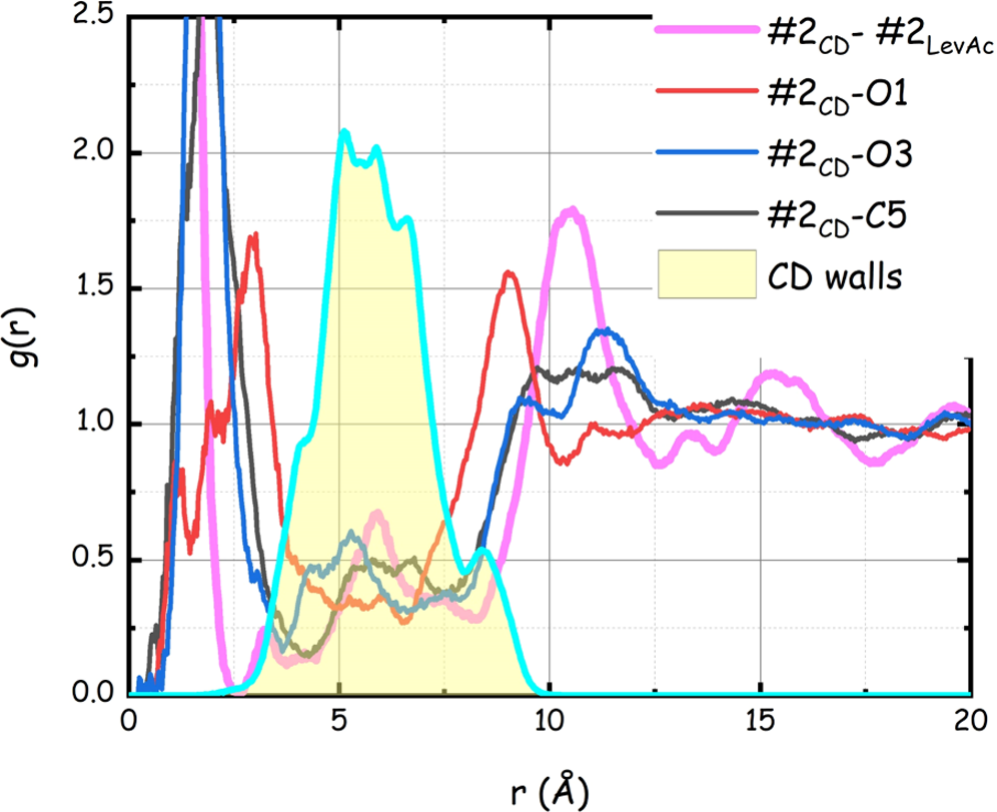
Selected MD-computed pair distribution functions between CD CoM (#2_CD_) and LevAc CoM (#2_LevAc_) and other relevant atoms. The shadowed area refers to the intramolecular pdf between CD CoM and all of the other CD atoms.

The nature of intermolecular interactions between SUPRA-DES components can be best appreciated by accessing different correlation functions extracted from the MD simulation. Figure 3 reports selected pair distribution functions (pdf, *g*(*r*)) between the CD center of mass (CoM, #2) and the LevAc CoM and other LevAc atoms (see Scheme 1; other relevant pdfs are shown in Figure S2). In the figures, the yellow-shadowed area refers to the pdf between CD CoM and all of the other CD atoms in the macromolecule in order to facilitate the distinction between correlations inside and outside of the CD walls, respectively. Different features emerge from the inspection of the two figures. DiMeβ-CD is characterized by walls extending up to ca. 10 Å from the CD CoM. Inside these walls, one can detect the presence of LevAc (magenta curve): in particular, the integration of the curve #2_CD_–#2_LevAc_ (up to *r* = 3 Å) leads to the assessment of the average presence of one LevAc molecule inside the CD. At a further distance, for 3 < *r* (Å) < 8, one can appreciate the limited presence of LevAc close to the CD walls, most probably approaching the CD from its open sides. Finally, at larger distances, *r* > 8 Å, one can detect the characteristic solvation shells of LevAc surrounding the reference CD. A distinct solvation shell of the LevAc CoM is detected at distances of 10.5 Å, and a second shell can be observed at ca. 15 Å. At the minimum (ca. 12.5 Å), an average of ca. 35 LevAc molecules constitute the first solvation shell for the reference CD. This solvating layer essentially maintains separated neighbor CDs, as their average distance amounts at ca. 14 Å (vide infra). The number of LevAc molecules surrounding a reference CD (*n* ∼35) is approximately corresponding to the stoichiometry of the mixture DiMeβ-CDs–LevAc (1:27), prompting that the SUPRA-DES stoichiometry might be determined not only by the hydrogen-bonding compatibility between the two compound families but also by the capability of LevAc to efficiently solvate and hence maintain neighbor CDs separated.

**Figure 4:**
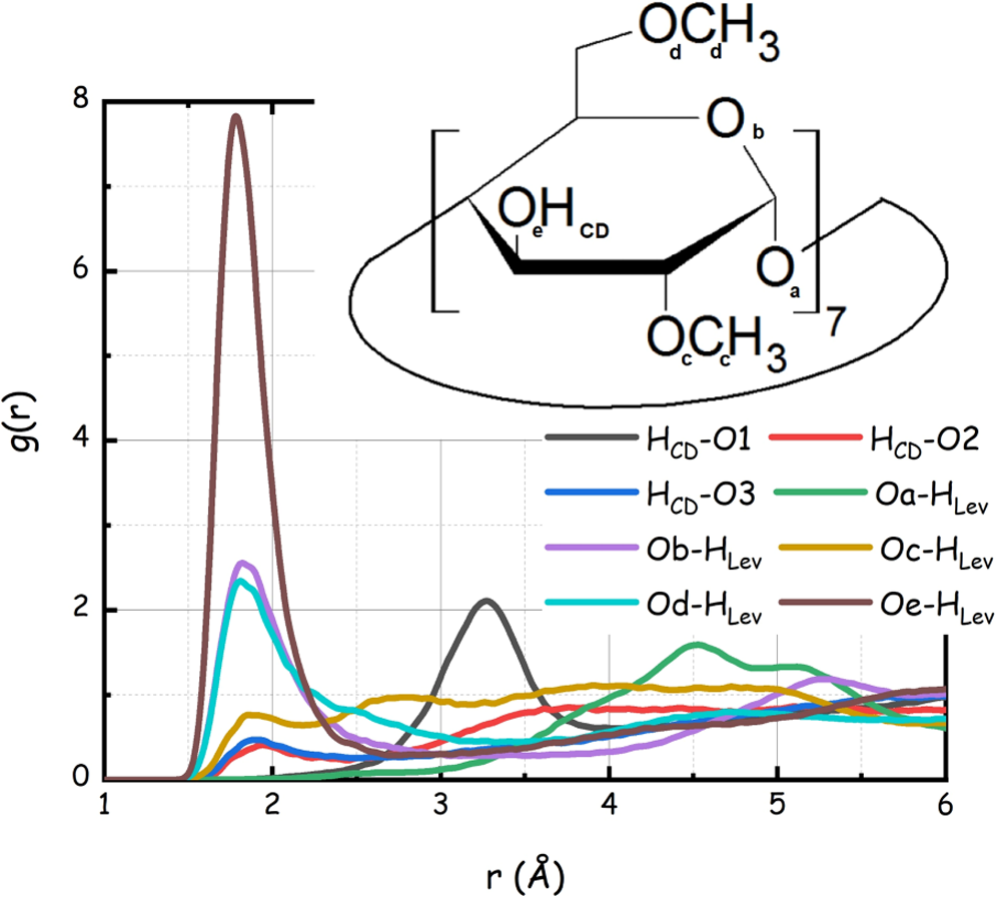
Selected MD-computed pair distribution functions related to hydrogen-bonding interactions between DiMeβ-CD and LevAc.

Additional inspection of Figure 3 (and Figure S2) allows extracting further information on the nature of LevAc coordination toward CD. The carboxyl group (oxygen atoms O1 and O2 and carbon atom C1) approaches CD external walls at the closest distance, most likely engaging in HB interactions with the CD rims. Next, carbon C2 and oxygen O3 from LevAc can approach the CD, while carbon C5, belonging to the methyl group, shows only a weak correlation. In view of these results prompting for specific correlations between CD and the HB active moieties in LevAc, we further explored the role of hydrogen-bonding correlations in this system. DiMeβ-CD is characterized by the presence of seven HB donor sites and 35 HB acceptor ones; analogously, LevAc is characterized by a carboxylic group that can act as both HB acceptor (O1 and O2) and donor (H5) and the keto oxygen (O3) can act as an HB acceptor site. Overall then, the two species have a variety of options accessible for the development of an extended HB network, despite the methyl capping of 2/3 of the HB donor sites available in natural β-CD, leading to DiMeβ-CD. Figure 4 shows the pdfs relative to potential HB active site correlations between DiMeβ-CD and LevAc (for the CD atom nomenclature, please refer to Scheme 1 or to the inset of Figure 4). One can observe that short-range interactions with pdf amplitude larger than one are found for CD’s Ob, Od, and Oe donor sites with the H_Lev_ atom (corresponding to H5) of LevAc only. Correlations between CD’s HB donor atoms (H_CD_) and LevAc oxygen O2 and O3 are short but occur with very low amplitude, while negligible correlation occurs with O1. These data then deliver a structural scenario, where LevAc acts as a strong HB donor agent toward DiMeβ-CD HB acceptor sites, while its HB acceptor capability toward DiMeβ-CD is very limited. This is also a consequence of the fact that >90% of the CD’s HB donor atoms (H_CD_) are involved in intramolecular HB interactions with the neighbor methoxy groups, involving Oc (see Figure S3), hence a negligible intermolecular engagement of methoxy oxygen atom Oc into HB interactions with LevAc HB donor units.

Such intra-CD HB interactions are relatively short; however, they tend to show an average angle O–H···O of the order of 15° and appear to deviate from linear, with respect to other less restricted intermolecular HB interactions, such as the ones between LevAc H_Lev_ and CD’s HB acceptor sites. This is shown in the Supporting Information (Figure S4a–d), where the combined distribution functions for either intra-CD or intermolecular CD–LevAc HB interactions are shown. The intermolecular distribution functions describing the geometry of HB interactions involving H_Lev_ and CD’s HB acceptor sites Oe, Ob, and Od are shown in the Supporting Information (Figure S4b–d, respectively). Therein, one can observe a larger angular distribution for the interactions involving Ob and Od as compared with the one involving the Oe hydroxyl group (Figure S4b). This is mainly due to the additional possibility of the bifurcated coordination of H_Lev_ by both Ob and Od, together with the more conventional direct coordination of either Ob or Od toward H_Lev_. Overall, the HB network topology is recollected in the Sankey plot reported in Figure S5: therein, the elevated HB donor capability of LevAc toward both CD and LevAc HB acceptor sites is clear, while only limited HB donor activity is exerted by CD.

The methyl groups belonging to the CD methoxy moieties can also engage in interactions with LevAc. In particular, we monitored the correlations between CD’s methyl Cc and Cd carbon atoms and the different C (C1, ···, C5) and O (O1, O2, O3) sites in LevAc (see Scheme 1); the corresponding pdfs and running coordination numbers are reported in the Supporting Information (Figure S6). From the inspection of these data, one can draw the conclusion that both methoxy carbon atoms (Cc and Cd) are efficiently surrounded by LevAc C5 and C1 atoms, the methyl group and the carboxyl group carbon atoms, respectively. The former group, however, approaches the methoxy carbons at the closest distance. Considering the CD Cc carbon, whose methoxy oxygen is engaged in intra-CD HB interactions, it is found that it is involved in a concerted solvation by LevAc C5, C1, O1, and O2. Such a dispersive interaction is not directly related to HB correlations. The solvation of Cd is somehow different. The neighbor oxygen, Od, is not involved in intra-CD HB correlations and together with Ob is involved in intermolecular HB interactions with LevAc. We find a concerted coordination of Cd by C5 and C1 carbon atoms belonging to LevAc, similarly to what was detected in the upper rim. In the present case, however, we also detect the occurrence of a synergic coordination of LevAc toward two different methoxy groups of the reference CD. In the Supporting Information (Figure S7), we show that an HB interaction between the LevAc carboxyl group with a CD methoxy group occurs in a synergic way with an interaction between the LevAc methyl group and the methyl group belonging to a different methoxy group of the same CD. Accordingly, we can conclude that rim solvation in the present system can get established due to the concurrent existence of HB-mediated correlations and dispersive interactions.

**Figure 5:**
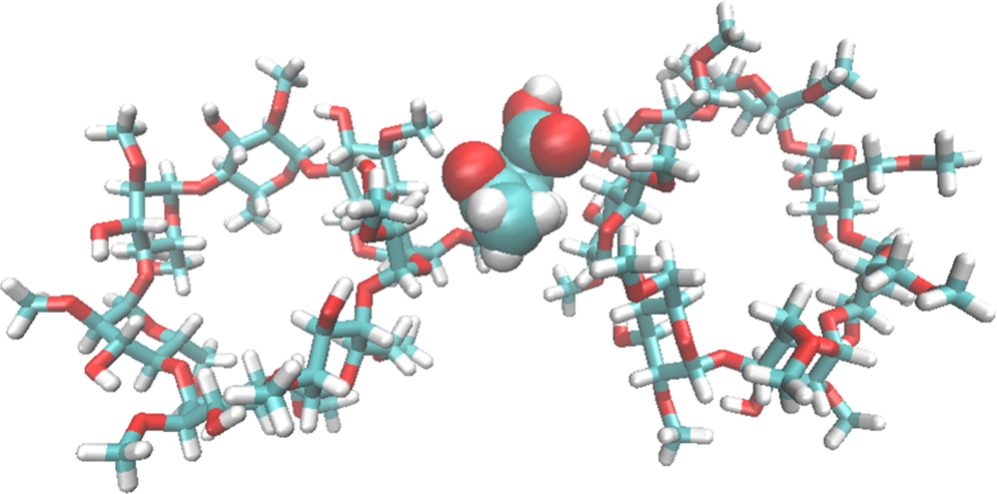
Representative configuration extracted from the MD simulation representing two neighbor DiMeβ-CDs (liquorice representation) that are separated by a LevAc molecule (CPK representation) whose C4 carbon atom is less than 6 Å apart from the center of a glucose ring of the two CDs.

We next explore the nature of solvation of the hydrophobic external walls of DiMeβ-CD. We determined the pseudo-spatial distribution functions of different LevAc moieties around CD’s walls (obtained by the isotropic averaging of atom distributions around the vertical CD symmetry axis, as a function of the distance from the axis) (see Figure S8). These figures show that LevAc carbon atoms C1 and C2 (data reported in Figure S8a,b) and the corresponding oxygen atoms bound to them (O1–3; data reported in Figure S8f–h) are distributed very close to the hydrogen-bonding acceptor sites located at the CD rims. On the other hand, the CD walls are approached by the CH_2_ and CH_3_ groups of LevAc (C3, C4, and C5; data reported in Figure S8c–e). This finding suggests that dispersive interactions are responsible for the solvation of this apolar CD portion. Inspection of simulation snapshots (such as the one reported in the Supporting Information (Figure S1)) indicates that, due to the high concentration of the mixture, DiMeβ-CDs are quite close to each other, although not aggregated. On average, the mutual distance between the first neighbor CDs is of the order of 14 Å; for comparison,^18^ β-CDs dissolved in the DES reline showed a first neighbor distance of ca. 20 Å (we notice that the latter mixture contained ca. half of the CD content than the present SUPRA-DES). The situation is nevertheless largely different from the case of flocculating β-CDs dissolved in water, where large clusters of juxtaposing CDs are detected and the first neighbor distance is ca. 10 Å (unpublished data). The intricate SUPRA-DES structural scenario (the similar RAMEB–LevAc SUPRA-DES has 10 times higher viscosity than neat LevAc at 40 °C^30^) is characterized by the presence of approaching CDs that are separated by a thin layer of LevAc molecules. Such a situation is shown in Figure 5, where the two first neighbor DiMeβ-CDs are separated by a LevAc molecule whose C4 carbon atom is less than 6 Å apart from the center of a glucose ring of the two CDs. It is noteworthy that the shown LevAc molecule is not engaged in hydrogen-bonding interactions with either CD molecules and only a dispersive interaction occurs between these neighbor molecules.

This structural scenario reflects the intricate mingling of HB and dispersive interactions in establishing the homogeneous liquid state of the DiMeβ-CD–LevAc. It is well known that the mere establishment of HB correlations between CDs and solvent cannot guarantee stable CD solutions and the role of dispersive interactions in hindering the hydrophobic coalescence of CD walls has been often highlighted.^18,63,66^ This study reveals that such a feature is also responsible for the occurrence of highly concentrated CD environments, such as SUPRA-DES. In Table S1, we show a decomposition of interaction energies between CD and LevAc, in terms of Coulombic and dispersive interactions. Therein, it emerges that while Coulombic interactions are dominant in influencing, especially DiMeβ-CD/DiMeβ-CD and LevAc/LevAc correlations, nonetheless, dispersive interactions contribute largely to such correlations but even turn out to be dominant in influencing DiMeβ-CD/LevAc correlations. Hence, the synergic interplay between electrostatic, hydrogen-bonding, and dispersive interactions dictates the overall structural scenario in a complex fluid such as the present SUPRA-DES and determines its variegated solvation capability toward polar, apolar, and amphiphilic compounds. This delicate cooperative action toward solvation is responsible for the enhanced SUPRA-DES characteristics in blending green and sustainable features with efficient supramolecular solvation capability, thus paving the way for safe, low-cost, and effectual chemical processing.

## Conclusions

An emerging class of solvents, with supramolecular properties and low melting behavior, has been recently introduced and referred to as SUPRA-DES. Binary mixtures of functionalized CDs and hydrogen-bonding donor agents, such as levulinic acid, are archetypal examples of such a class. They are characterized by a glassy behavior upon cooling that prevents detecting their melting point and leads to a wide liquid window where these solvents can be efficiently exploited. The CD capability of forming inclusion complexes with hydrophobic compounds and the polar environment generated by the extended hydrogen-bonding network in bulk make the SUPRA-DES an “extremely multiactive mixture”^33^ with its capacity to both engage polar molecules in bulk and encapsulate apolar compound in the hydrophobic CD cavity. This supramolecular, complex architecture can pave the way to numerous smart applications in the near future, provided details in the nature of these media are fully perceived. In this study, we dealt with the structural organization in the archetypal SUPRA-DES composed of DiMeβ-CD and LevAc at a ratio of 1:27. We preliminarily probed the mesoscopic morphology of such a system, by means of SAXS, verifying that no CD aggregation occurs in this highly concentrated system and CDs are homogeneously distributed in bulk. Next, the microscopic organization of LevAc around CD has been probed by MD simulations. First, we verified the paramount role played by the intermolecular HB interaction in CD solvation. This interaction, however, is not the only one active in the system, and strong synergies between HB and dispersive interactions have been observed, leading to the capability of LevAc to efficiently screen hydrophobic correlations between neighbor CD and hence preventing their collapse. Overall, this first study on the structure of a SUPRA-DES can provide a useful guide on how to unleash the enormous potential of this new class of solvent media for sustainable development.

## Supporting Information Available

Additional figures, including MD simulation box snapshots, different pdfs, different cdfs, Sankey plot describing HB topology and pseudo-sdf on the solvation of CD by LevAc moieties, and a table with the decomposition of interaction energies in terms of Coulombic and dispersive correlations (PDF)
Force field parameters used for GROMACS simulations (ZIP)

## References

[ref1] Williams G. T.; Haynes C. J. E.; Fares M.; Caltagirone C.; Hiscock J. R.; Gale P. A. Advances in Applied Supramolecular Technologies. Chem. Soc. Rev. 2021, 50, 2737–2763. 10.1039/D0CS00948B. 33438685

[ref2] Lehn J.-M. Perspectives in Supramolecular Chemistry—From Molecular Recognition towards Molecular Information Processing and Self-Organization. Angew. Chem., Int. Ed. 1990, 29, 1304–1319. 10.1002/anie.199013041.

[ref3] Lehn J.-M. Toward Complex Matter: Supramolecular Chemistry and Self-Organization. Proc. Natl. Acad. Sci. U.S.A. 2002, 99, 4763–4768. 10.1073/pnas.072065599. 11929970 PMC122664

[ref4] Chen G.; Jiang M. Cyclodextrin-Based Inclusion Complexation Bridging Supramolecular Chemistry and Macromolecular Self-Assembly. Chem. Soc. Rev. 2011, 40, 2254 10.1039/c0cs00153h. 21344115

[ref5] Yu G.; Jie K.; Huang F. Supramolecular Amphiphiles Based on Host–Guest Molecular Recognition Motifs. Chem. Rev. 2015, 115, 7240–7303. 10.1021/cr5005315. 25716119

[ref6] Ma X.; Zhao Y. Biomedical Applications of Supramolecular Systems Based on Host-Guest Interactions. Chem. Rev. 2015, 115, 7794–7839. 10.1021/cr500392w. 25415447

[ref7] Morin-Crini N.; Fourmentin S.; Fenyvesi É.; Lichtfouse E.; Torri G.; Fourmentin M.; Crini G. 130 Years of Cyclodextrin Discovery for Health, Food, Agriculture, and the Industry: A Review. Environ. Chem. Lett. 2021, 19, 2581–2617. 10.1007/s10311-020-01156-w.

[ref8] Crini G.; Fourmentin S.; Fenyvesi É.; Torri G.; Fourmentin M.; Morin-Crini N. Cyclodextrins, from Molecules to Applications. Environ. Chem. Lett. 2018, 16, 1361–1375. 10.1007/s10311-018-0763-2.

[ref9] Szejtli J. Introduction and General Overview of Cyclodextrin Chemistry. Chem. Rev. 1998, 98, 1743–1754. 10.1021/cr970022c. 11848947

[ref10] Connors K. A. The Stability of Cyclodextrin Complexes in Solution. Chem. Rev. 1997, 97, 1325–1357. 10.1021/cr960371r. 11851454

[ref11] Rekharsky M. V.; Inoue Y. Complexation Thermodynamics of Cyclodextrins. Chem. Rev. 1998, 98, 1875–1917. 10.1021/cr970015o. 11848952

[ref12] He Y.; Li P.; Yalkowsky S. H. Solubilization of Fluasterone in Cosolvent/Cyclodextrin Combinations. Int. J. Pharm. 2003, 264, 25–34. 10.1016/S0378-5173(03)00389-2. 12972333

[ref13] Kfoury M.; Geagea C.; Ruellan S.; Greige-Gerges H.; Fourmentin S. Effect of Cyclodextrin and Cosolvent on the Solubility and Antioxidant Activity of Caffeic Acid. Food Chem. 2019, 278, 163–169. 10.1016/j.foodchem.2018.11.055. 30583356

[ref14] Li P.; Zhao L.; Yalkowsky S. H. Combined Effect of Cosolvent and Cyclodextrin on Solubilization of Nonpolar Drugs. J. Pharm. Sci. 1999, 88, 1107–1111. 10.1021/js990159d. 10564056

[ref15] Nakhle L.; Kfoury M.; Greige-Gerges H.; Fourmentin S. Effect of Dimethylsulfoxide, Ethanol, α- and β-Cyclodextrins and Their Association on the Solubility of Natural Bioactive Compounds. J. Mol. Liq. 2020, 310, 113156 10.1016/j.molliq.2020.113156.

[ref16] Fourmentin S.; Landy D.; Moura L.; Tilloy S.; Bricout H. H.; Ferreira M. Procédé d’épuration d’un Effluent Gazeux. France Patent FR3058905B1, 2016.

[ref17] McCune J. A.; Kunz S.; Olesińska M.; Scherman O. A. DESolution of CD and CB Macrocycles. Chem. – Eur. J. 2017, 23, 8601–8604. 10.1002/chem.201701275. 28339123

[ref18] Triolo A.; Lo Celso F.; Russina O. Structural Features of β-Cyclodextrin Solvation in the Deep Eutectic Solvent, Reline. J. Phys. Chem. B 2020, 124, 2652–2660. 10.1021/acs.jpcb.0c00876. 32097007

[ref19] Di Pietro M. E.; Colombo Dugoni G.; Ferro M.; Mannu A.; Castiglione F.; Costa Gomes M.; Fourmentin S.; Mele A. Do Cyclodextrins Encapsulate Volatiles in Deep Eutectic Systems?. ACS Sustainable Chem. Eng. 2019, 7, 17397–17405. 10.1021/acssuschemeng.9b04526.

[ref20] Di Pietro M. E.; Castiglione F.; Mele A. Polar/Apolar Domains’ Dynamics in Alkylimidazolium Ionic Liquids Unveiled by the Dual Receiver NMR 1H and 19F Relaxation Experiment. J. Mol. Liq. 2021, 322, 114567 10.1016/j.molliq.2020.114567.

[ref21] Moufawad T.; Moura L.; Ferreira M.; Bricout H.; Tilloy S.; Monflier E.; Costa Gomes M.; Landy D.; Fourmentin S. First Evidence of Cyclodextrin Inclusion Complexes in a Deep Eutectic Solvent. ACS Sustainable Chem. Eng. 2019, 7, 6345–6351. 10.1021/acssuschemeng.9b00044.

[ref22] Abbott A. P.; Capper G.; Davies D. L.; Rasheed R. K.; Tambyrajah V. Novel Solvent Properties of Choline Chloride/Urea Mixtures. Chem. Commun. 2003, 1, 70–71. 10.1039/B210714G. 12610970

[ref23] Smith E. L.; Abbott A. P.; Ryder K. S. Deep Eutectic Solvents (DESs) and Their Applications. Chem. Rev. 2014, 114, 11060–11082. 10.1021/cr300162p. 25300631

[ref24] Martins M. A. R.; Pinho S. P.; Coutinho J. A. P. Insights into the Nature of Eutectic and Deep Eutectic Mixtures. J. Solution Chem. 2019, 48, 962–982. 10.1007/s10953-018-0793-1.

[ref25] Ferreira M.; Jérôme F.; Bricout H.; Menuel S.; Landy D.; Fourmentin S.; Tilloy S.; Monflier E. Rhodium Catalyzed Hydroformylation of 1-Decene in Low Melting Mixtures Based on Various Cyclodextrins and N,N′-Dimethylurea. Catal. Commun. 2015, 63, 62–65. 10.1016/j.catcom.2014.11.001.

[ref26] Florindo C.; Lima F.; Ribeiro B. D.; Marrucho I. M. Deep Eutectic Solvents: Overcoming 21st Century Challenges. Curr. Opin. Green Sustainable Chem. 2019, 18, 31–36. 10.1016/j.cogsc.2018.12.003.

[ref27] Paiva A.; Craveiro R.; Aroso I.; Martins M.; Reis R. L.; Duarte A. R. C. Natural Deep Eutectic Solvents – Solvents for the 21st Century. ACS Sustainable Chem. Eng. 2014, 2, 1063–1071. 10.1021/sc500096j.

[ref28] Zhang Q.; De Oliveira Vigier K.; Royer S.; Jérôme F. Deep Eutectic Solvents: Syntheses, Properties and Applications. Chem. Soc. Rev. 2012, 41, 7108–7146. 10.1039/c2cs35178a. 22806597

[ref29] El Achkar T.; Moufawad T.; Ruellan S.; Landy D.; Greige-Gerges H.; Fourmentin S. Cyclodextrins: From Solute to Solvent. Chem. Commun. 2020, 56, 3385–3388. 10.1039/D0CC00460J. 32100798

[ref30] El Achkar T.; Moura L.; Moufawad T.; Ruellan S.; Panda S.; Longuemart S.; Legrand F.-X.; Costa Gomes M.; Landy D.; Greige-Gerges H.; Fourmentin S. New Generation of Supramolecular Mixtures: Characterization and Solubilization Studies. Int. J. Pharm. 2020, 584, 119443 10.1016/j.ijpharm.2020.119443. 32447025

[ref31] El Masri S.; Ruellan S.; Zakhour M.; Auezova L.; Fourmentin S. Cyclodextrin-Based Low Melting Mixtures as a Solubilizing Vehicle: Application to Non-Steroidal Anti-Inflammatory Drugs. J. Mol. Liq. 2022, 353, 118827 10.1016/j.molliq.2022.118827.

[ref32] Abranches D. O.; Coutinho J. A. P. Type V Deep Eutectic Solvents: Design and Applications. Curr. Opin. Green Sustainable Chem. 2022, 35, 100612 10.1016/j.cogsc.2022.100612.

[ref33] Janicka P.; Kaykhaii M.; Płotka-Wasylka J.; Gębicki J. Supramolecular Deep Eutectic Solvents and Their Applications. Green Chem. 2022, 24, 5035–5045. 10.1039/D2GC00906D.

[ref34] Li G.; Jiang Y.; Liu X.; Deng D. New Levulinic Acid-Based Deep Eutectic Solvents: Synthesis and Physicochemical Property Determination. J. Mol. Liq. 2016, 222, 201–207. 10.1016/j.molliq.2016.07.039.

[ref35] Deng D.; Han G.; Jiang Y. Investigation of a Deep Eutectic Solvent Formed by Levulinic Acid with Quaternary Ammonium Salt as an Efficient SO2 Absorbent. New J. Chem. 2015, 39, 8158–8164. 10.1039/c5nj01629k.

[ref36] Maugeri Z.; Domínguez De María P. Novel Choline-Chloride-Based Deep-Eutectic-Solvents with Renewable Hydrogen Bond Donors: Levulinic Acid and Sugar-Based Polyols. RSC Adv. 2012, 2, 421–425. 10.1039/c1ra00630d.

[ref37] Gutiérrez A.; Zamora L.; Benito C.; Atilhan M.; Aparicio S. Insights on Novel Type V Deep Eutectic Solvents Based on Levulinic Acid. J. Chem. Phys. 2022, 156, 094504 10.1063/5.0080470. 35259877

[ref38] El Masri S.; Masri S. El.; Ruellan S.; Zakhour M.; Auezova L. Cyclodextrin-Based Low Melting Mixtures as a Solubilizing Vehicle: Application to Non-Steroidal Anti-Inflammatory Drugs. J. Mol. Liq. 2022, 353, 118827 10.1016/j.molliq.2022.118827.

[ref39] Petitprez J.; Xavier F.; Catherine L.; Pipkin T. J. D.; Antle V.; Kfoury M.; Fourmentin S. Huge Solubility Increase of Poorly Water - Soluble Pharmaceuticals by Sulfobutylether - β - Cyclodextrin Complexation in a Low - Melting Mixture. Environ. Chem. Lett. 2022, 20, 1561–1568. 10.1007/s10311-022-01415-y.

[ref40] Guo H.; Li L.; Xu X.; Zeng M.; Chai S.; Wu L.; Li H. Semi-Solid Superprotonic Supramolecular Polymer Electrolytes Based on Deep Eutectic Solvents and Polyoxometalates. Angew. Chem., Int. Ed. 2022, 61, e202210695 10.1002/anie.202210695. 36106475

[ref41] Kfoury M.; Landy D.; Fourmentin S. Combination of DES and Macrocyclic Host Molecules: Review and Perspectives. Curr. Opin. Green Sustainable Chem. 2022, 36, 100630 10.1016/j.cogsc.2022.100630.

[ref42] Farooq M. Q.; Zeger V. R.; Anderson J. L. Comparing the Extraction Performance of Cyclodextrin-Containing Supramolecular Deep Eutectic Solvents versus Conventional Deep Eutectic Solvents by Headspace Single Drop Microextraction. J. Chromatogr. A 2021, 1658, 462588 10.1016/j.chroma.2021.462588. 34662824

[ref43] Zhang J.; Yao L.; Li S.; Li S.; Wu Y.; Li Z.; Qiu H. Green Materials with Promising Applications: Cyclodextrin-Based Deep Eutectic Supramolecular Polymers. Green Chem. 2023, 10.1039/D3GC00489A.

[ref44] Hammond O. S.; Bowron D. T.; Edler K. J. Liquid Structure of the Choline Chloride-Urea Deep Eutectic Solvent (Reline) from Neutron Diffraction and Atomistic Modelling. Green Chem. 2016, 18, 2736–2744. 10.1039/C5GC02914G.

[ref45] Kaur S.; Kumari M.; Kashyap H. K. Microstructure of Deep Eutectic Solvents: Current Understanding and Challenges. J. Phys. Chem. B 2020, 124, 10601–10616. 10.1021/acs.jpcb.0c07934. 33151072

[ref46] Busato M.; Del Giudice A.; Di Lisio V.; Tomai P.; Migliorati V.; Gentili A.; Martinelli A.; D’Angelo P. Fate of a Deep Eutectic Solvent upon Cosolvent Addition: Choline Chloride-Sesamol 1:3 Mixtures with Methanol. ACS Sustainable Chem. Eng. 2021, 9, 12252–12261. 10.1021/acssuschemeng.1c03809. 34552826 PMC8442355

[ref47] Hess B.; Kutzner C.; van der Spoel D.; Lindahl E. GROMACS 4: Algorithms for Highly Efficient, Load-Balanced, and Scalable Molecular Simulation. J. Chem. Theory Comput. 2008, 4, 435–447. 10.1021/ct700301q. 26620784

[ref48] Van Der Spoel D.; Lindahl E.; Hess B.; Groenhof G.; Mark A. E.; Berendsen H. J. C. GROMACS: Fast, Flexible, and Free. J. Comput. Chem. 2005, 26, 1701–1718. 10.1002/jcc.20291. 16211538

[ref49] Jorgensen W. L.; Maxwell D. S.; Tirado-Rives J. Development and Testing of the OPLS All-Atom Force Field on Conformational Energetics and Properties of Organic Liquids. J. Am. Chem. Soc. 1996, 118, 11225–11236. 10.1021/ja9621760.

[ref50] Doherty B.; Acevedo O. OPLS Force Field for Choline Chloride-Based Deep Eutectic Solvents. J. Phys. Chem. B 2018, 122, 9982–9993. 10.1021/acs.jpcb.8b06647. 30125108

[ref51] Cézard C.; Trivelli X.; Aubry F.; Djedaïni-Pilard F.; Dupradeau F. Y. Molecular Dynamics Studies of Native and Substituted Cyclodextrins in Different Media: 1. Charge Derivation and Force Field Performances. Phys. Chem. Chem. Phys. 2011, 13, 15103–15121. 10.1039/c1cp20854c. 21792425

[ref52] Gebhardt J.; Kleist C.; Jakobtorweihen S.; Hansen N. Validation and Comparison of Force Fields for Native Cyclodextrins in Aqueous Solution. J. Phys. Chem. B 2018, 122, 1608–1626. 10.1021/acs.jpcb.7b11808. 29287148

[ref53] Zhang H.; Ge C.; Van Der Spoel D.; Feng W.; Tan T. Insight into the Structural Deformations of Beta-Cyclodextrin Caused by Alcohol Cosolvents and Guest Molecules. J. Phys. Chem. B 2012, 116, 3880–3889. 10.1021/jp300674d. 22376204

[ref54] Zhang H.; Tan T.; Feng W.; Van Der Spoel D. Molecular Recognition in Different Environments: β-Cyclodextrin Dimer Formation in Organic Solvents. J. Phys. Chem. B 2012, 116, 12684–12693. 10.1021/jp308416p. 23025718

[ref55] Martínez L.; Andrade R.; Birgin E. G.; Martínez J. M. PACKMOL: A Package for Building Initial Configurations for Molecular Dynamics Simulations. J. Comput. Chem. 2009, 30, 2157–2164. 10.1002/jcc.21224. 19229944

[ref56] Bussi G.; Donadio D.; Parrinello M. Canonical Sampling through Velocity Rescaling. J. Chem. Phys. 2007, 126, 014101 10.1063/1.2408420. 17212484

[ref57] Parrinello M.; Rahman A. Polymorphic Transitions in Single Crystals: A New Molecular Dynamics Method. J. Appl. Phys. 1981, 52, 7182–7190. 10.1063/1.328693.

[ref58] Darden T.; York D.; Pedersen L. Particle Mesh Ewald: An N·log(N) Method for Ewald Sums in Large Systems. J. Chem. Phys. 1993, 98, 10089–10092. 10.1063/1.464397.

[ref59] Essmann U.; Perera L.; Berkowitz M. L.; Darden T.; Lee H.; Pedersen L. G. A Smooth Particle Mesh Ewald Method. J. Chem. Phys. 1995, 103, 8577–8593. 10.1063/1.470117.

[ref60] Brehm M.; Kirchner B. TRAVIS - A Free Analyzer and Visualizer for Monte Carlo and Molecular Dynamics Trajectories. J. Chem. Inf. Model. 2011, 51, 2007–2023. 10.1021/ci200217w. 21761915

[ref61] Hollóczki O.; Macchiagodena M.; Weber H.; Thomas M.; Brehm M.; Stark A.; Russina O.; Triolo A.; Kirchner B. Triphilic Ionic-Liquid Mixtures: Fluorinated and Non-Fluorinated Aprotic Ionic-Liquid Mixtures. ChemPhysChem 2015, 16, 3325–3333. 10.1002/cphc.201500473. 26305804 PMC4641458

[ref62] Brehm M.; Thomas M.; Gehrke S.; Kirchner B. TRAVIS—A Free Analyzer for Trajectories from Molecular Simulation. J. Chem. Phys. 2020, 152, 164105 10.1063/5.0005078. 32357781

[ref63] Triolo A.; Celso F.; Lo Perez J.; Russina O. Solubility and Solvation Features of Native Cyclodextrins in 1-Ethyl-3-Methylimidazolium Acetate. Carbohydr. Polym. 2022, 291, 119622 10.1016/j.carbpol.2022.119622. 35698349

[ref64] Kusmin A.; Lechner R. E.; Kammel M.; Saenger W. Native and Methylated Cyclodextrins with Positive and Negative Solubility Coefficients in Water Studied by SAXS and SANS. J. Phys. Chem. B 2008, 112, 12888–12898. 10.1021/jp802031w. 18798665

[ref65] Jeffries C. M.; Graewert M. A.; Blanchet C. E.; Langley D. B.; Whitten A. E.; Svergun D. I. Preparing Monodisperse Macromolecular Samples for Successful Biological Small-Angle X-Ray and Neutron-Scattering Experiments. Nat. Protoc. 2016, 11, 2122–2153. 10.1038/nprot.2016.113. 27711050 PMC5402874

[ref66] Loftsson T.; Saokham P.; Sá Couto A. R. Self-Association of Cyclodextrins and Cyclodextrin Complexes in Aqueous Solutions. Int. J. Pharm. 2019, 560, 228–234. 10.1016/j.ijpharm.2019.02.004. 30771468

