# Supplementary material for: Liquid Structure Scenario of the Archetypal Supramolecular Deep Eutectic Solvent: Heptakis(2,6-di-O-methyl)-β-cyclodextrin/levulinic Acid
Source: ACS Sustain Chem Eng. 2023 Jun 2;11(24):9103–10. doi: 10.1021/acssuschemeng.3c01858 (PMC10283020; doi:10.1021/acssuschemeng.3c01858)
Supplement: Supplementary file 1 — sc3c01858_si_001.pdf [file sc3c01858_si_001.pdf]

Supporting Information.

Liquid structure scenario of the archetypal supramolecular deep eutectic solvent: Heptakis(2,6-di-O-methyl)-β-cyclodextrin/levulinic acid.

Alessandro Triolo[1,*], Fabrizio Lo Celso[1,2], Sophie Fourmentin[3], and Olga Russina[1,4,*]

[1] Laboratorio Liquidi Ionici, Istituto Struttura della Materia, Consiglio Nazionale delle Ricerche (ISM-CNR), Rome 00133, Italy

[2] Department of Physics and Chemistry, Università di Palermo, Palermo, Italy

[3] Unité de Chimie Environnementale et Interactions sur le Vivant (UCEIV, UR 4492), Université du Littoral Côte d'Opale (ULCO), Dunkerque, France

[4] Department of Chemistry, University of Rome Sapienza, Rome 00185, Italy

Corresponding Authors:

Alessandro Triolo (triolo@ism.cnr.it);

Olga Russina (olga.russina@uniroma1.it)

Number of pages: 10

Number of Figures: 8

Number of Tables: 1

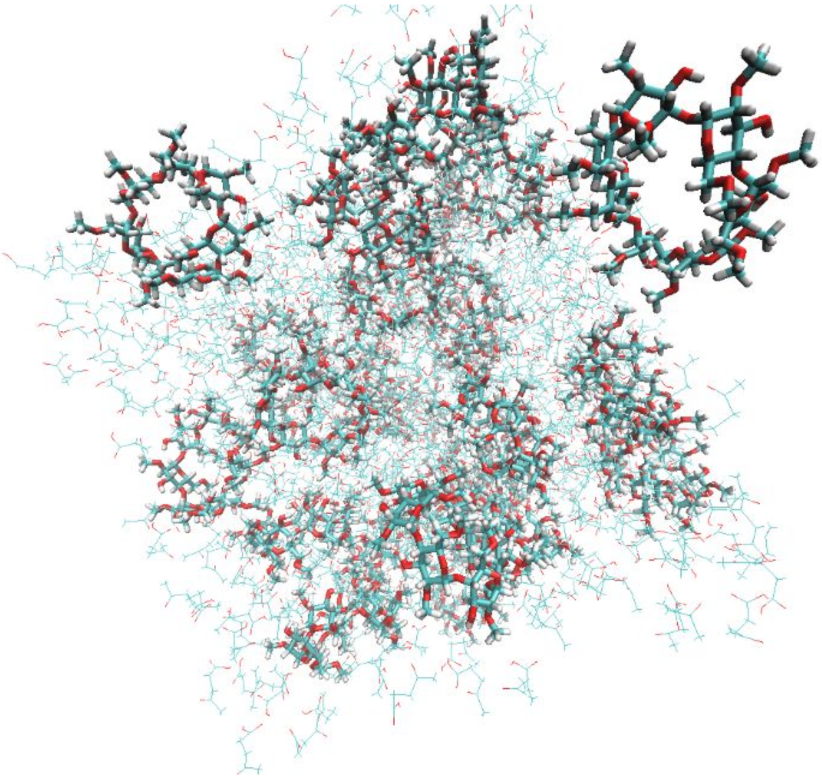

Figure S1. Representative snapshot of the studied DiMeβ-CD – Levulinic acid SUPRA-DES, where the homogeneous distribution of CDs is highlighted across the Levulinic acid matrix.

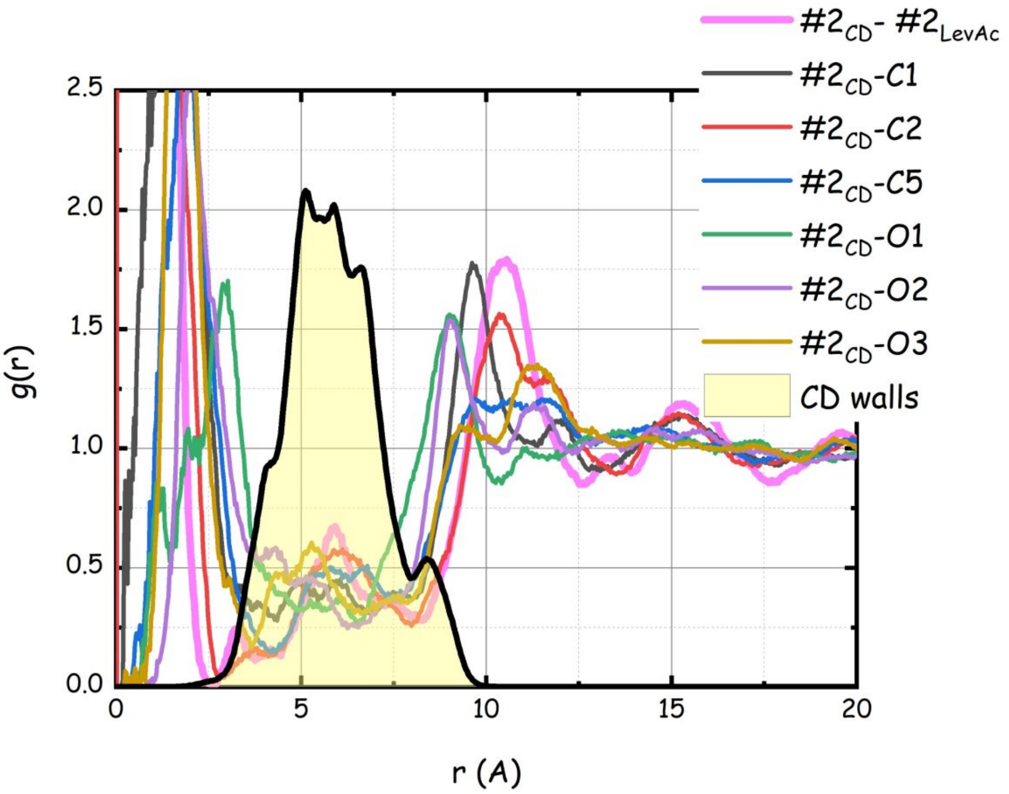

Figure S2. MD-computed pair distribution functions between CD CoM and Levulinic acid CoM and other relevant atoms. The shadowed area refers to the pdf between CD CoM and all the other CD atoms.

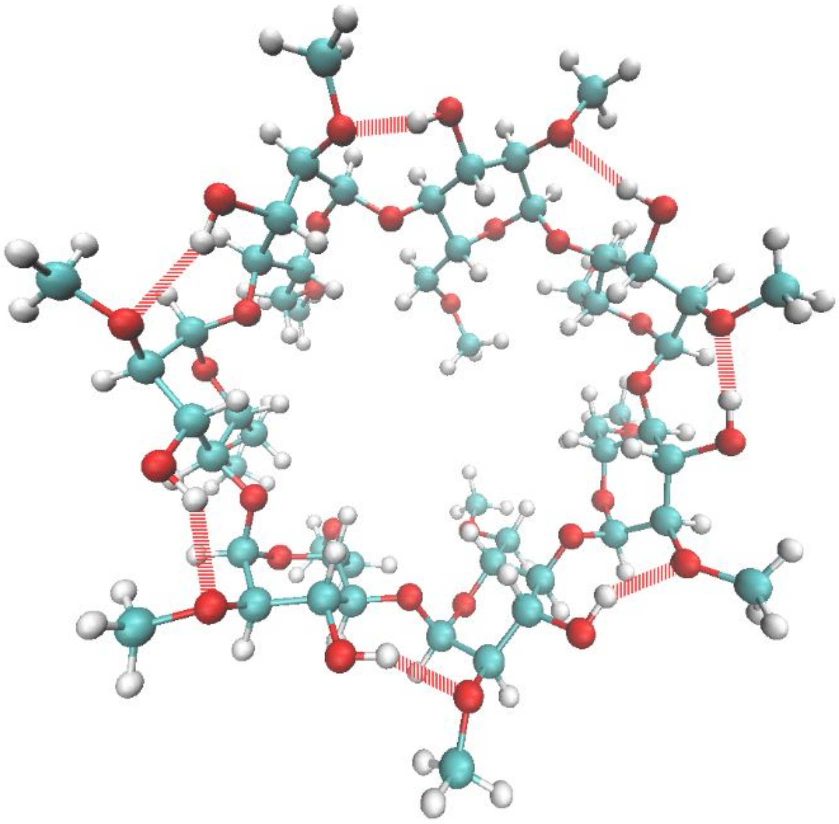

Figure S3. Description of a representative DiMeβ-CD molecule in the SUPRADES and the observed intra molecular hydrogen bonding interactions between methoxy and hydroxyl groups in position 2 and 3, respectively. The HB interactions satisfy the geometric conditions of $d_{O\cdots O}$< 3.2 Å and $angle_{O\text{-}H\cdots O}$<37°.

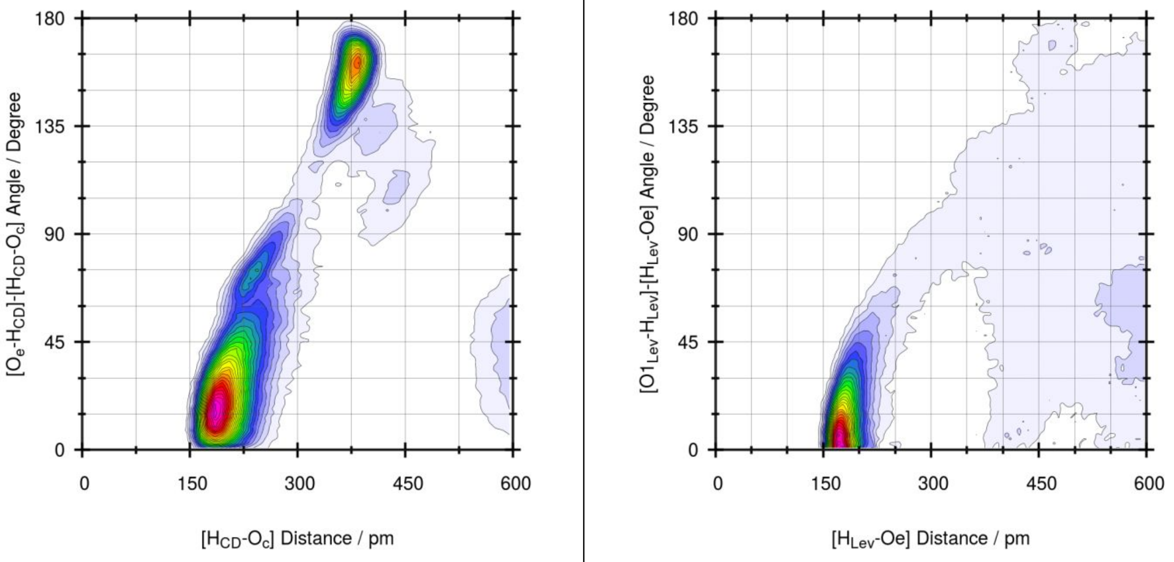

a) **b)**

Figure S4. a) CDF for DiMeβ-CD intramolecular HB interactions between hydroxyl proton and methoxy oxygen; b) CDF for intermolecular HB interaction between LevAc carboxyl hydrogen H_{Lev} and CD hydroxyl oxygen, Oe.

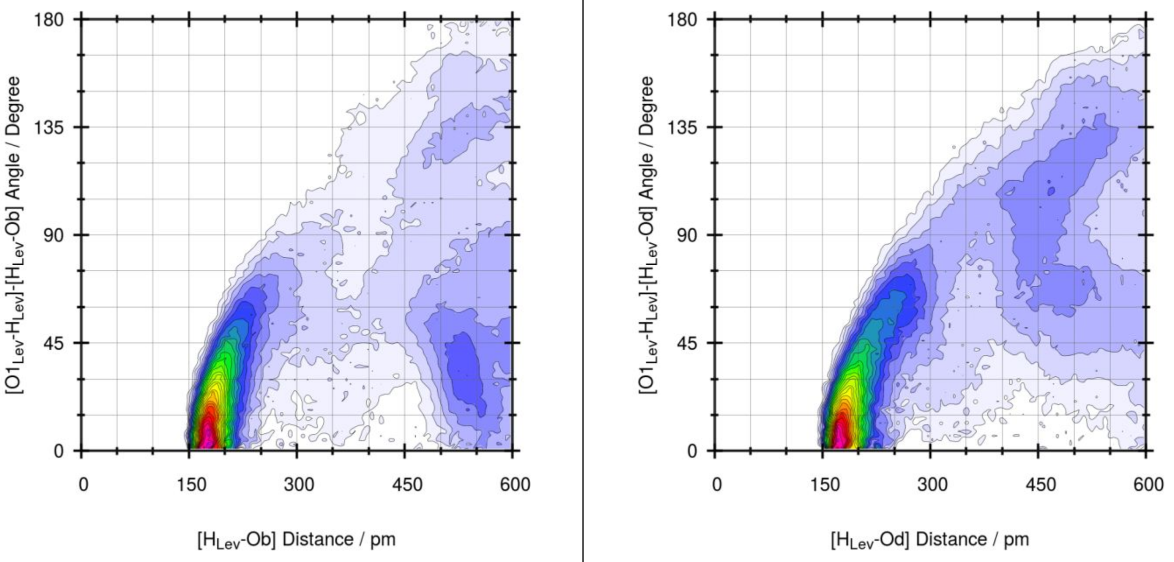

c) **d)**

Figure S4. c) CDF for HB interactions between LevAc carboxyl proton and CD Ob site; d) CDF for HB interaction between LevAc carboxyl hydrogen and CD Od site.

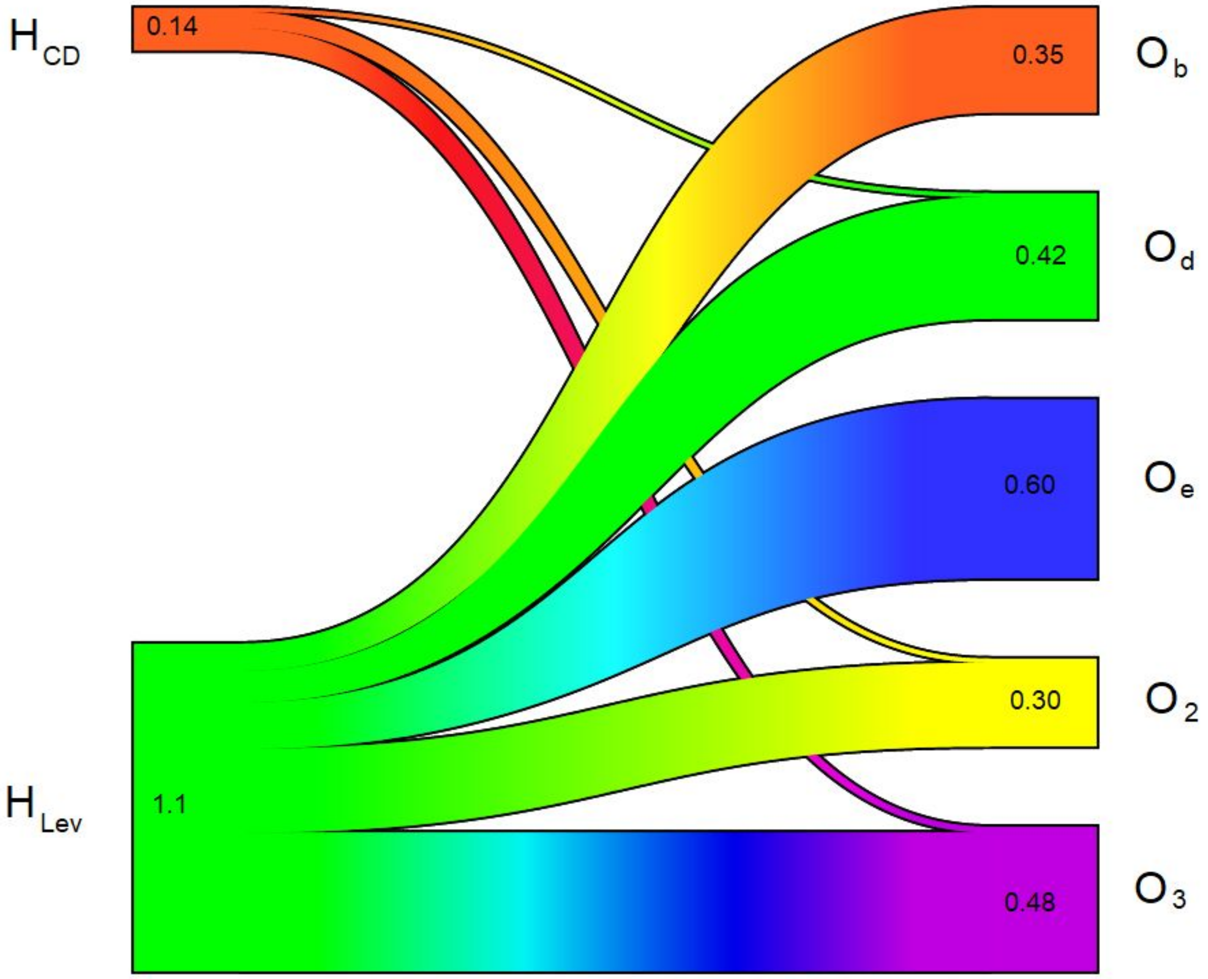

Figure S5. Sankey plot describing the topology of the HB network in the DiMeβ-CD/LevAc SUPRA-DES. HB donors H’s are reported in the left side of the diagram, while HB acceptors are shown in the right side. Ob, Od and Oe refer to CD oxygen atoms, while O2 and O3 refer to LevAc oxygen atoms. . The number indicate the average HB count per atom (atomic nomenclature as indicated in Scheme 1).

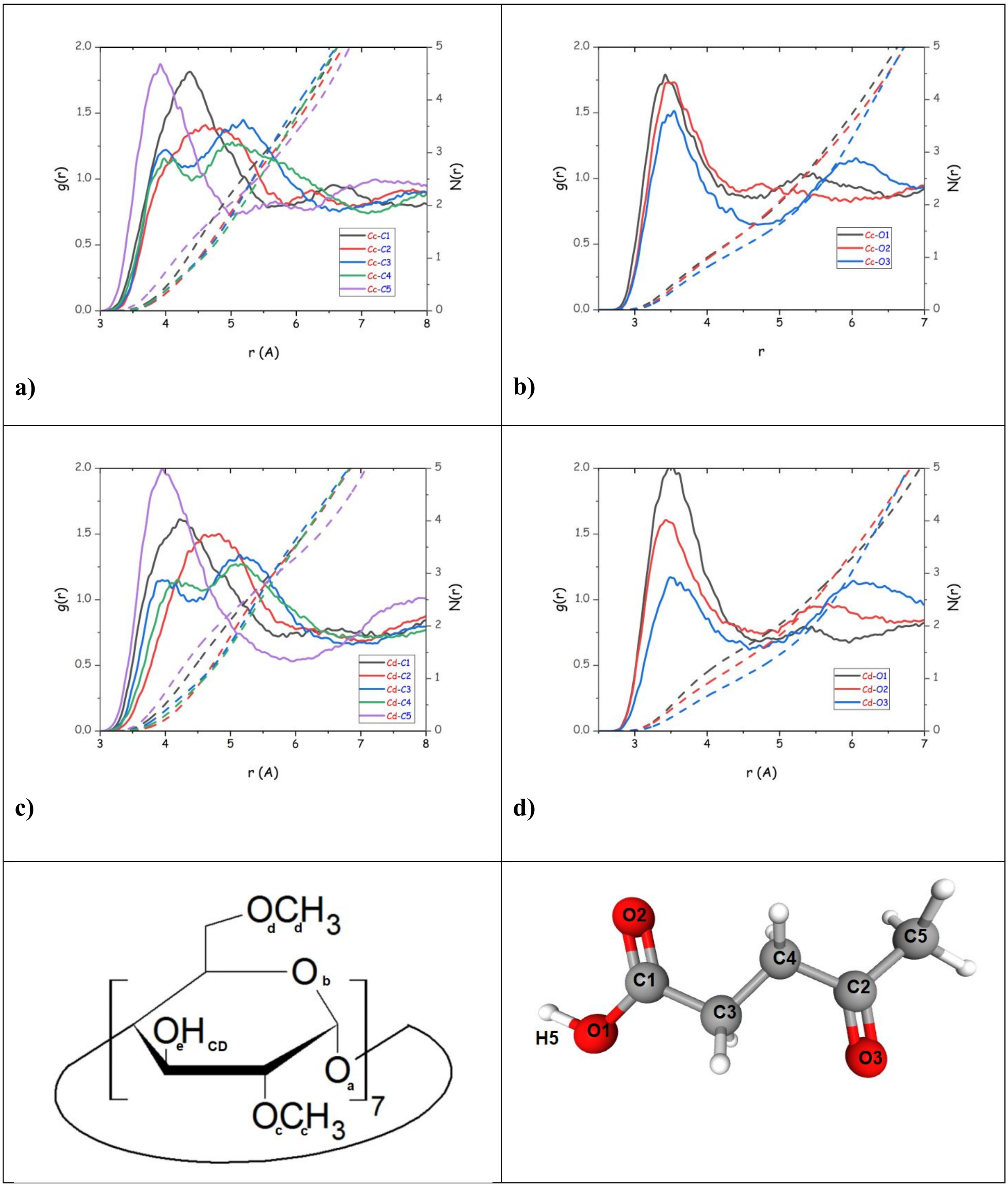

Figure S6. Pair distribution (continuous lines) and running coordination numbers (dashed lines) functions describing the interaction between CD's methoxy carbons, Cc (upper panels) and Cd (lower panel) and Levulinic acid carbons (C1-C5) (left panels) and oxygen (O1-O3) (right panels) atoms. In the bottom panels, Scheme 1 is reported to follow atomic nomenclature.

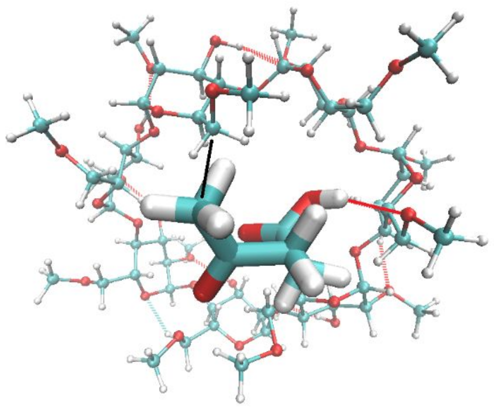

Figure S7. Description of a representative snapshot where CD's methoxy Carbon Cd is coordinated by the levulinic acid C5 atom (black line). It is shown that such a coordination is concerted with an hydrogen bonding mediated interaction between Levulinic acid carboxyl group and a CD neighbour methoxy group (red line).

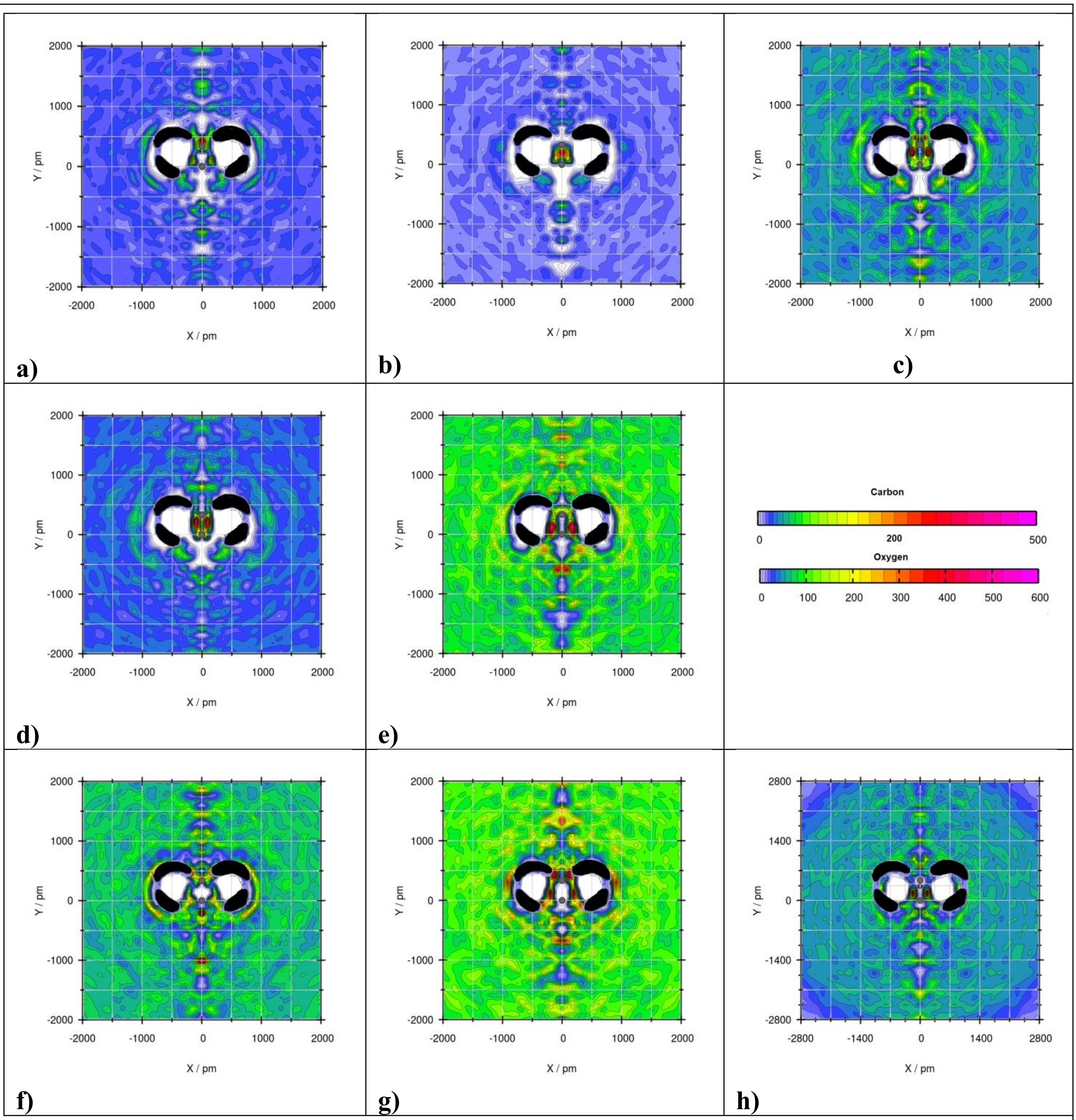

Figure S8. Pseudo spatial distribution functions describing the distribution of different LevAc moieties with respect to a reference DiMeβ-CD vertical axis. The different LevAc moieties are: a) C1; b) C2; c) C3; d) C4; e) C5; f) O1; g) O2; h) O3. The colour scales are reported in the central right panel for the carbon (top) and the oxygen (bottom) species. The blackened areas refer to the distribution of the intramolecular CD hydroxyl and methoxy groups.

Pair	Coulombic Interaction (kJ/mol)	Dispersive Interaction (kJ/mol)
DiMeβ-CD - DiMeβ-CD	-27999.8	-7677.4
DiMeβ-CD -LevAc	-11521.2	-22083.3
LevAc-LevAc	-158848.0	-37626.6

Table S1. Decomposition of total interaction energies between different chemical pairs (810 LevAc and 30 DiMeβ-CD) in terms of Coulombic and dispersive correlations.
